# Consumption of crustaceans by megaherbivorous dinosaurs: dietary flexibility and dinosaur life history strategies

**DOI:** 10.1038/s41598-017-11538-w

**Published:** 2017-09-21

**Authors:** Karen Chin, Rodney M. Feldmann, Jessica N. Tashman

**Affiliations:** 1 Department of Geological Sciences and Museum of Natural History, University of Colorado Boulder, Boulder, CO 80309 USA; 2 Department of Geology, Kent State University, Kent, OH 44242 USA

## Abstract

Large plant-eating dinosaurs are usually presumed to have been strictly herbivorous, because their derived teeth and jaws were capable of processing fibrous plant foods. This inferred feeding behavior offers a generalized view of dinosaur food habits, but rare direct fossil evidence of diet provides more nuanced insights into feeding behavior. Here we describe fossilized feces (coprolites) that demonstrate recurring consumption of crustaceans and rotted wood by large Late Cretaceous dinosaurs. These multi-liter coprolites from the Kaiparowits Formation are primarily composed of comminuted conifer wood tissues that were fungally degraded before ingestion. Thick fragments of laminar crustacean cuticle are scattered within the coprolite contents and suggest that the dinosaurian defecators consumed sizeable crustaceans that sheltered in rotting logs. The diet of decayed wood and crustaceans offered a substantial supply of plant polysaccharides, with added dividends of animal protein and calcium. Nevertheless, it is unlikely that the fossilized fecal residues depict year-round feeding habits. It is more reasonable to infer that these coprolites reflected seasonal dietary shifts—possibly related to the dinosaurs’ oviparous breeding activities. This surprising fossil evidence challenges conventional notions of herbivorous dinosaur diets and reveals a degree of dietary flexibility that is consistent with that of extant herbivorous birds.

## Introduction

In the 1800s, the earliest dinosaur workers used tooth morphology to conclude that newly-discovered iguanodontid and hadrosaurid dinosaurs fed on vegetation^1, 2^. Leidy^2^ suggested that *Hadrosaurus* was “…a vegetable feeding Reptile, one which masticated its food like the herbivorous Mammalia”. Since then, reconstructions of the feeding behaviors of large ornithischians have been largely based on extant mammalian megaherbivores^3^ which can be regarded as body-size analogs. Body size is relevant to diet because large extant herbivores tend to have lower quality diets than smaller plant-eaters^4, 5^. More detailed analyses have corroborated early deductions that ornithischian tooth and skull morphologies are consistent with fibrous plant diets^6–8^. Other features such as palaeobotanical context^9–11^ and inferred physiology^12^ have augmented generalized interpretations of ornithischian diets.

Nevertheless, specific information about ornithischian food choices is scant. Large non-avian dinosaurs have no extant physiological or phylogenetic analogs, and direct evidence for herbivorous dinosaur diets is rare. Fossilized digestive residues from herbivores are not only uncommon, but are difficult to recognize and attribute to specific organisms. Fortunately a small number of cases of direct fossil evidence for diet allow us to test our inferences about the feeding behavior of large herbivorous dinosaurs. The discoveries of reasonably well-substantiated gut contents within the articulated carcasses of an ankylosaur^13^, a brachylophosaur^14^, and a neornithischian^15^ indicate that plant reproductive tissues and leafy browse were indeed ingested by ornithischians. Such dietary choices are comparable to the feeding habits of extant mammalian browsers. However, coprolite evidence from Montana points to more dietary complexity by demonstrating that some ornithischians periodically fed on large quantities of rotting wood^16^. We report here the discovery of a new assemblage of wood-filled coprolites that not only indicates that wood consumption was far-ranging, but reveals another unexpected feeding habit of herbivorous dinosaurs—recurring ingestion of sizeable crustaceans. These specimens challenge us to reevaluate some of our preconceived perceptions of dinosaur feeding behavior.

## Specimens and Contents

**Figure 1:**
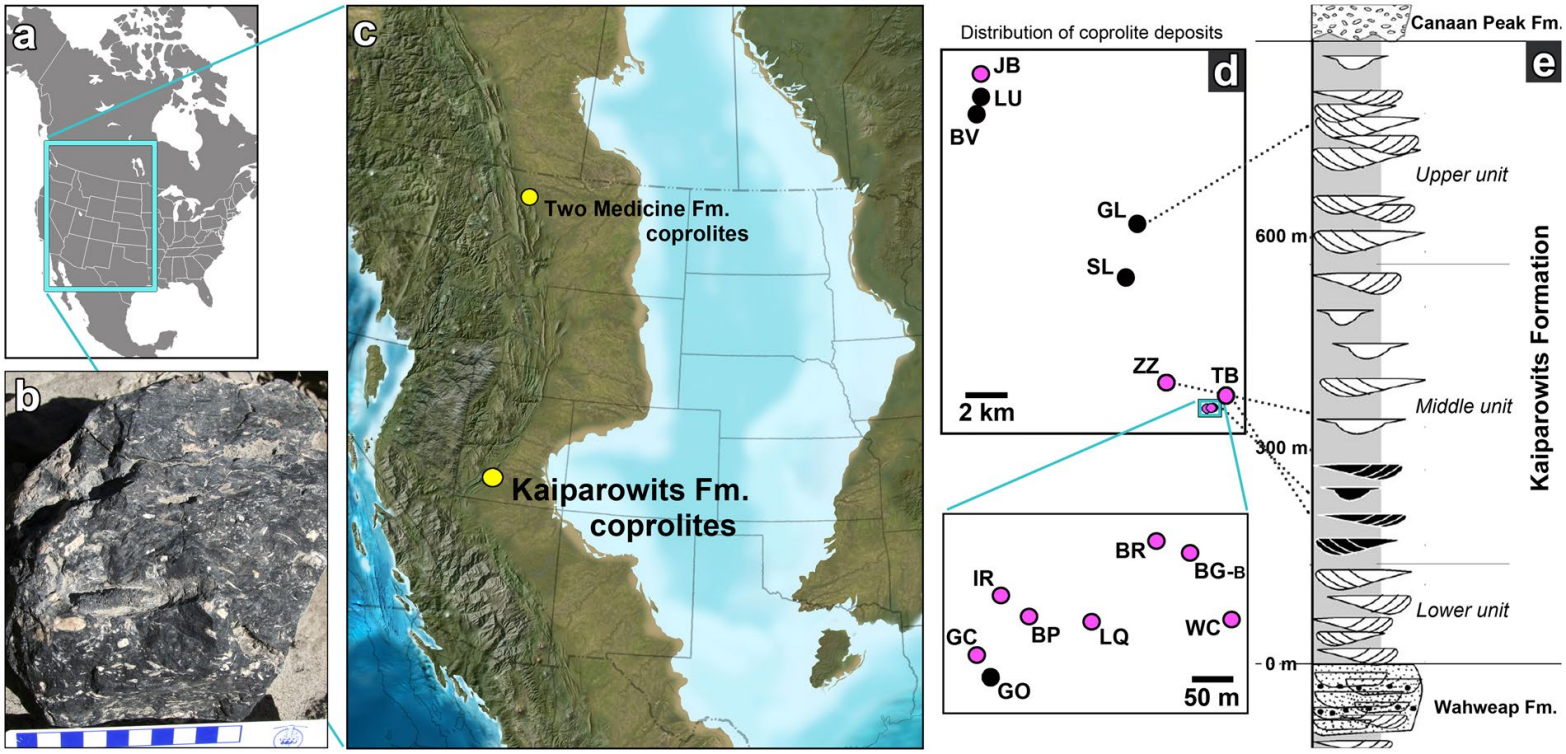
Geographic and stratigraphic setting of coprolites in the Kaiparowits Formation. (**a**) North American map with blue box indicating area of map in (**c**). (**b**) Coprolite in the field with characteristic dark colour and backfilled burrow characteristic of dung beetle activity. (**c**) Reconstruction of western North America during the Middle Campanian, ~77 mya. The Kaiparowits Fm. is ~1000 km south of the Two Medicine Fm. Image modified from Blakey R, Paleogeography of the Western Interior Seaway of North America © 2014 Colorado Plateau Geosystems Inc. (**d**) Upper box showing widespread distribution of coprolite deposits in the Kaiparowits Fm. Pink dots indicate coprolite deposits with discernable crustacean cuticle evident in coprolites. Dotted lines point to approximate positions of some coprolite deposits within the stratigraphic column in (**e**). Lower box shows area with eight coprolite deposits represented by small blue rectangle in the upper box. (**e**) Stratigraphic column illustrating generalized alluvial architecture of the Kaiparowits Fm. Black sand bodies indicate zone that appears to be tidally-influenced. Stratigraphic column from Roberts^18^ and Lawton *et al*.^72^ with consent from Elsevier and SEPM (Society for Sedimentary Geology). This figure is not covered by the CC BY licence. All rights reserved, used with permission.

The new coprolite deposits were found in the Kaiparowits Formation of Grand Staircase-Escalante National Monument in southern Utah (Fig. 1). This thick (~860 m) sedimentary package was deposited between ~76.0–74.1 million years ago^17^ on an alluvial plain interlaced with meandering rivers and ponds^18^. Most of the irregularly-shaped coprolites are exposed in deflation lags of overbank deposits, however two *in situ* specimens were found within uncemented, fine-grained sediments as discrete blocky masses (not in lens-shaped beds). Because exposed coprolites are typically broken into numerous pieces, a coprolite deposit is defined as one to multiple coprolite pieces that appear to have derived from a single localized area. The sizes of the exposed deposits are variable, ranging from sub decimeter-sized fragments, to multi-decimeter blocks, to masses that cover several square meters and appear to represent multiple defecation events. More than 15 discrete coprolite deposits were discovered within at least three stratigraphic levels in the lower half of the middle unit of the Kaiparowits Formation (Eric Roberts pers. comm.) at sites up to 20 km apart (Fig. 1).

**Figure 2:**
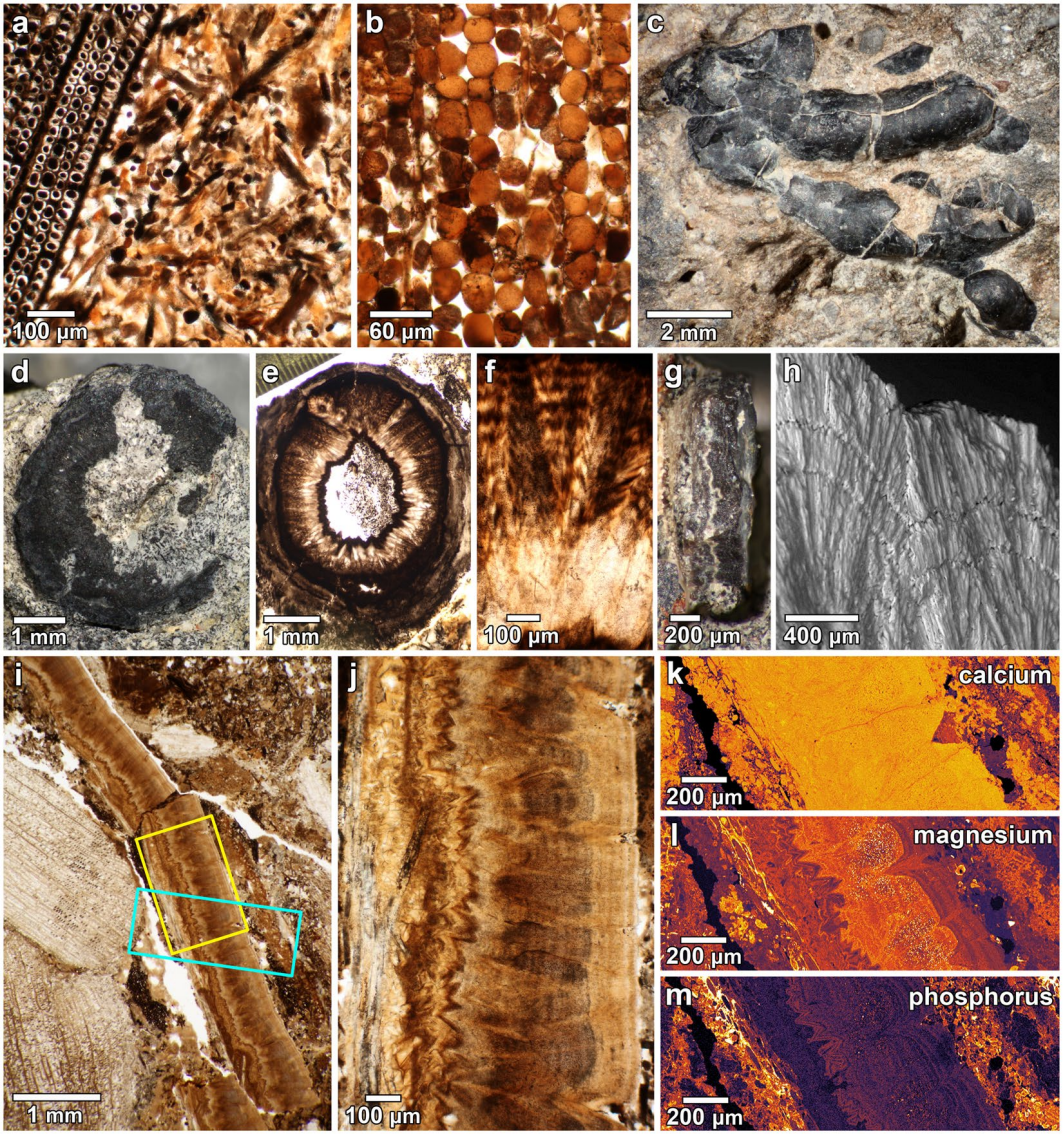
Coprolite contents. (**a**) Photomicrograph showing thin section of typical coprolite ground mass comprised of conifer wood fragments and disaggregated wood tracheids (DMNH EPV.98868; WC-13a-4c). (**b**) Photomicrograph of cross sectional view of a decayed wood fragment. The loss of cell walls and middle lamellae reveals selective delignification by white rot fungi (DMNH EPV.62494; BP-12-2c). (**c**) Surface of irregular, knobby cuticle (DMNH EPV.98868; WC-13a-1). (**d–f**) Piece of cylindrical appendage embedded in coprolitic ground mass (DMNH EPV.62494; BP-13b-17). (**e**) Thin section of appendage shown in (**d**) showing circular configuration and thick cuticle relative to appendage diameter. (**f**) Higher magnification photomicrograph of appendage cuticle in (**d**) showing diagenetically altered laminae. (**g-h**) Small cuticle fragment with internal laminae evident (DMNH EPV.62494; BP-12-7). (**h**) Scanning electron micrograph of specimen in (**g**) revealing perpendicular diagenetic growth of crystals through the laminae. (**i-j**) Thin section (DMNH EPV.62494; BP-12-13f) showing a >6 mm long cuticle fragment embedded in fecal ground mass. Yellow rectangle indicates area shown in (**j**) and blue rectangle shows area of microprobe maps. (**j**) Close-up image of cuticle in (**i**) revealing conspicuous parallel laminae. Exocuticle is at right of photo and probable pores are evident. (**k**–**m**) Qualitative microprobe element maps showing distributions of calcium, magnesium, and phosphorus of cuticle in (**i**). Brighter colours indicate higher element concentrations. Note that distributions of magnesium and phosphorus follow the laminar structure of the cuticle.

Thin section analyses reveal that the dark, calcareous coprolites (Fig. 1b) are primarily comprised of jumbled fragments of conifer wood embedded within a groundmass of disaggregated wood tracheid cells and relatively few clastic grains (Fig. 2a,b). Open and backfilled burrows are common in the fecal deposits. The backfilled burrows often show a meniscate fill, and demonstrate that the burrowers actively translocated organic material within or beneath the fecal masses. These fossil burrows resemble the burrowing traces of extant dung beetles which lay eggs in burrows provisioned with dung^19^. Thus the specimens are identified as coprolites on the basis of their comminuted organic contents, paucity of clastic grains, pervasive backfilled burrows, sedimentological context, and similarity to 17 previously-described coprolite deposits from the Two Medicine Formation^16, 20^.

At least 10 of 15 examined coprolite deposits contain fragments of dark, shell-like material. The relative abundance of this material in different coprolite samples is variable, and diagenetically-altered fragments are often very difficult to distinguish from wood. However, macroscopic and petrographic analyses suggest that the shell-like fragments commonly comprise up to 5% of coprolite specimens. The dark shell fragments are variable in appearance, exhibiting a range of preservational states that likely reflect both digestive and diagenetic processes. Well-preserved external surfaces can be smooth, nodose, or knobby (Fig. 2c). Most fragments are flat to gently curved, and are up to ~2 × 3 cm in surface area. Other pieces are tubular, indicating cylindrical appendages (Fig. 2d,e). Thin sections of well-preserved fragments range from ~0.5 to 1 mm in thickness and show internal parallel laminae and vertical channels that may be pores (Fig. 2e,f,i,j). Qualitative WDS electron microprobe elemental maps of a section of well-preserved cuticle show a predominance of calcium (Fig. 2k) with trace distributions of magnesium (Fig. 2l) and phosphorus (Fig. 2m) that follow the internal laminar structure (WDS maps of eight elements mapped on this thin section are available in the Supplementary Microprobe Maps S1). Most fragments show diagenetic alteration in the form of crystal growth perpendicular to the laminar structure of the shell-like material. The progression of recrystallization is revealed by scanning electron micrographs that show crystal prisms growing through the parallel laminae (Fig. 2h).

Mollusk shell is also evident in at least six of the 13 examined coprolite deposits. Some of the shell is highly fragmented, and may have been ingested. However, it is likely that at least some of the gastropods in the coprolites had sheltered and fed within dung pats that were already deposited. This is supported by the discovery of several intact, sub-millimeter to 2 mm sized gastropods in the coprolites, suggesting breeding within the fecal masses. Similar post-depositional colonization of dinosaur feces by gastropods was observed in the Two Medicine coprolites^21^.

## Discussion

No direct evidence links these Kaiparowits coprolites with specific producers, however, the large size and woody contents suggest that the fecal masses were produced by large herbivorous dinosaurs with dentition that could process fibrous diets. Because sympatric congeners often display niche partitioning^7, 22^, it is likely that most or all of the wood-bearing coprolites were produced by the same or similar dinosaur taxa. Several large ornithischian taxa have been recovered from this unit. Hadrosaur bones are most common^23^, including those from the lambeosaurine *Parasaurolophus* and two species of the hadrosaurine *Gryposaurus*. Three ceratopsid taxa^24^, an ankylosaurid, nodosaurid, pachycephalosaurid, and a hypsilophondontid^23, 24^ have also been found in the Kaiparowits Formation. Of these, the larger bodied hadrosaurs and ceratopsians are good candidates to have produced the sizeable woody fecal masses in the Kaiparowits Formation. Both possessed multi-toothed dental batteries, but the crushing and shearing abilities of the dentition of hadrosaurs would have allowed them to effectively exploit a broader range of foods than the shearing teeth and jaws of ceratopsians^25^. Faunal context suggests that the similar coprolites from the Two Medicine Formation were likely produced by *Maiasaura* hadrosaurs^16, 20^, so hadrosaurs may have also been responsible for the coprolites from the Kaiparowits Formation.

### Interpretation of contents

It can be difficult to identify comminuted and diagenetically-altered food items within coprolites, but histological analyses of the inclusions in these specimens are revealing. The ubiquitous dissociated tracheids and other histological features reveal wood decay by white rot fungi. These delignifying fungi can degrade wood by either simultaneously attacking both lignin and cellulose, or by selectively destroying lignin. Because lignin helps bind individual tracheary elements together, selective delignification of wood destroys cell walls while leaving cellulose-rich tracheids intact, but detached from each other^26, 27^. Thus the abundant, disaggregated or loosely associated tracheids in the coprolites indicate that the wood was selectively delignified by white rot fungi. Since depolymerization of lignin requires aerobic conditions^28^, delignification of the wood could not have taken place inside a vertebrate gut, and would have occurred before ingestion. These observations suggest that the fecal masses in the Kaiparowits Formation were produced by dinosaurs that fed on selectively delignified, rotting logs. This feeding behavior is comparable to that indicated by the coprolites from the Two Medicine Formation^16^.

**Figure 3:**
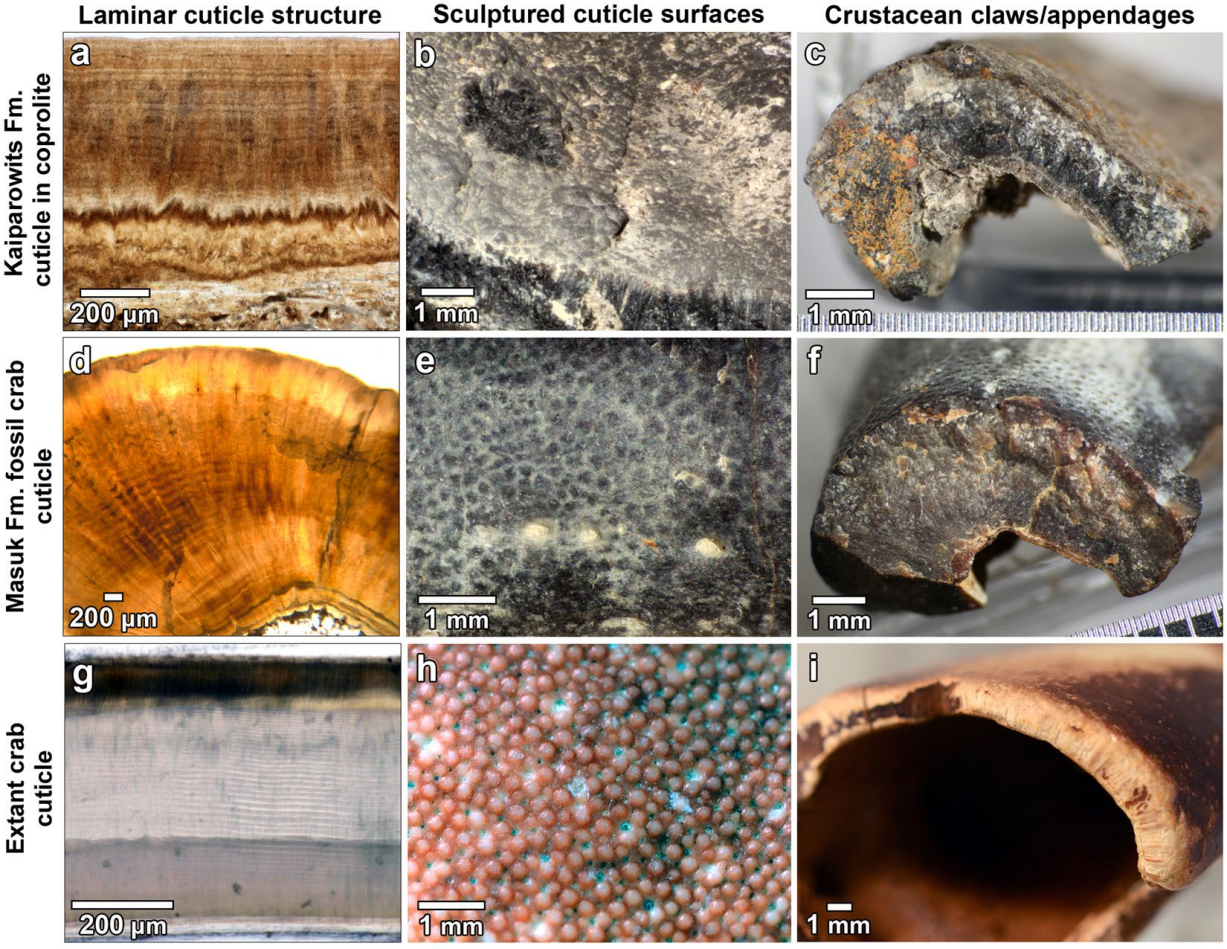
Cuticle features of Kaiparowits crustacean, fossil crab from the Masuk Formation, and extant crab specimens. (**a**–**c**) Cuticle from Kaiparowits coprolites. (**a**) Thin section showing laminae in well-preserved cuticle fragment. Dark structures at bottom of the image are disaggregated tracheids (specimen DMNH EPV.62494; BP-12-13g). (**b**) Side and top view of tuberculate cuticle surface (DMNH EPV.62494; BP-12-4). (**c**) Cross-sectional view of fragment of probable crustacean appendage. The curved configuration and asymmetrical cuticle thickness are characteristic of crustacean claws (DMNH EPV.624934; DP-13b-30). (**d**–**f**) Fossil crab cuticle from the Campanian Masuk Formation (specimens from the Sam Noble Oklahoma Museum of Natural History). (**d**) Thin section of claw cuticle showing parallel laminae. Note that cuticle is particularly thick through this section of the claw (specimen OU 238017-a). (**e**) Tuberculate surface of fossil claw (OU 238017). (**f**) Cross-sectional view of crab claw fragment showing asymmetry in cuticle thickness (OU 238019). (**g**–**i**) Cuticular features of recent and sub-recent crabs. (**g**) Thin section of laminar structure of *Callinectes* sp. cuticle. (**h**) Tuberculate surface of *Homolaspis plana* crab from Chile. (**i**) Cross sectional view of broken fragment of a sub-recent *Scylla serrata* crab claw from Guam.

However, the Kaiparowits coprolites are further distinguished by the pervasive fragmented shell-like inclusions. The ~0.9 mm thickness, smooth to nodose surfaces, and variable flexure of these rigid inclusions are all consistent with the external morphology of the exoskeletal material of crustaceans. Moreover, the calcareous composition and internal laminar structure are characteristic of crustacean cuticle, a calcified chitinous composite material characterized by fine laminae^29–31^. Equally telling is that some of the shell-like material is configured in tubular structures that signify appendages; the thickened cross-section and irregular lumen of a >4 mm diameter section of the material resembles the structure of extant decapod claws^32^ (Fig. 2d,e). These multiple, characteristic features of the shell-like inclusions in the Kaiparowits coprolites are comparable to those of both extant and fossil crustacean cuticle (Fig. 3).

We note that insect cuticle has a similar laminated structure, but is much thinner^33^ and far less mineralized^31^. Some of the rigid, shell-like inclusions also resemble fossil eggshell, however the irregular shell curvature, presence of appendages, and large-scale external ornamentation are at variance with eggshell. In addition, the distributions of magnesium and phosphorous described in extant eggshell^34^ are unlike the laminar distributions of magnesium and phosphorous evident in thin sections of the inclusions (Fig. 2l,m). We thus conclude that most or all of the distinctive shell-like laminar inclusions in the coprolites are fragments of crustacean cuticle.

Identification of the particular type of crustacean in the coprolites will require recovery of more diagnostic material. However, the large and thick pieces of cuticle, plus the presence of the 4 mm thick appendage suggest that these crustaceans were sizeable. Based on the presence of a 2 × 3 cm fragment of cuticle in one of the coprolites, we conservatively infer that a minimum dimension of the crustaceans was approximately 5 cm (with 2 cm added to account for the spread of paired appendages plus the missing carapace or tergite rims). Although the identity of the crustaceans in the coprolites is unknown, fossil crab claws have been recovered from the middle Campanian Wahweap Formation^35^ and its lateral equivalent, the Masuk Formation in eastern Utah^36^. These units are slightly older than the Kaiparowits Formation. Features of the crab fossils from the Masuk Formation are shown in Fig. 3.

Some of the cuticle fragments display prominent perpendicular crystals that are not present in unaltered crustacean cuticle. However, Hof and Briggs^37^ documented growth of calcium carbonate crystals within the cuticle of decaying mantis shrimps. Alteration by both digestion and diagenesis may have set the stage for cuticle recrystallization within the calcareous host sediments.

### Nutritional value of diet

The diet represented by the Kaiparowits coprolites would have provided a woody stew of plant, fungal, and invertebrate tissues. Selectively delignified wood offers carbon resources in the form of celluloses and hemicelluloses. Although extant megaherbivores are not known to feed on rotting wood, domesticated cattle in Chile actively consume delignified logs^27^. This feeding behavior is explained by studies that showed that the rumen digestibility of the Chilean woods increases from less than 4% in undecayed wood to 30–60% in delignified wood^38^. Subsequent analyses of fungally-degraded poplar wood has corroborated the enhanced rumen digestibility of delignified wood^39^.

Physical residues of fungal tissues are rare or absent in the coprolites, but the decayed state of the wood indicates that abundant fungal tissues were also ingested by the Kaiparowits fecal producers. The nutritional value of different fungal tissues is highly variable, however both reproductive^40^ and vegetative^41^ fungal tissues are known to augment the diets of a variety of extant vertebrates^40, 42^.

Although fragments of crustacean cuticle make up a relatively small proportion of the contents of the Utah coprolites, it is likely that moderate amounts of crustacean soft tissues were consumed. Relative to plants, most animal tissues contain higher percentages of balanced proteins, and are readily processed by autoenzymatic digestion^43, 44^. In addition, the calcareous crustacean cuticle would have offered substantial sources of calcium. Other invertebrate organisms would have almost certainly inhabited Late Cretaceous decaying logs, even though their tissues are not evident in the coprolites. Such fauna would have contributed supplemental high quality protein to the dinosaurs’ diets.

The occurrence of multiple coprolites containing an amalgam of wood, fungal, and invertebrate tissues from at least three stratigraphic levels in the Kaiparowits Formation indicates that this diet persisted through an extended period of time at this locality. Moreover, the similarity of the Kaiparowits coprolites to those from three stratigraphic levels in the Two Medicine Formation demonstrates that dinosaur taxa from different regions engaged in feeding on decaying wood in different habitats. It is notable that these two coeval formations are separated by at least 1,000 km and 10 degrees in latitude, and represent palaeoenvironments interpreted as wet and subtropical (Kaiparowits)^45^ versus more temperate (Two Medicine)^46^. Nevertheless, although the coprolite evidence indicates that consumption of delignified wood was widespread, this was probably not a year-round diet. Leaves and other herbaceous tissues are more typical herbivore fare, and regenerate much more rapidly than woody structural tissues. Thus it is unlikely that the two Cretaceous, coprolite-yielding habitats could have sustained populations of large dinosaurs that fed exclusively upon rotting wood. It is more plausible that these coprolites represent a periodic dietary switch by herbivorous dinosaurs that consumed other plant tissues during most of the year. The paucity of other types of herbivore coprolites from the Kaiparowits or other Cretaceous deposits is probably due to taphonomic biases; woody fecal masses would have had greater preservation potential than fecal residues containing less refractory contents.

### Incentives for feeding on rotting wood

Although feeding on rotted wood may have occurred on a seasonal or periodic basis, the documentation of 32 examples of wood-filled coprolite deposits (15 from the Kaiparowits and 17 from the Two Medicine^16^ formations) indicates that this recurring feeding behavior persisted through different generations and in different regions. Because the consumption of rotting wood and invertebrates has not been reported for extant megaherbivores, different hypotheses can be proposed to explain this unexpected dinosaur diet. Motivation to engage in this feeding behavior could have been driven by food scarcity or by nutritional opportunism.

Analysis of the Late Cretaceous Kaiparowits environment provides some context with which to evaluate these competing hypotheses. Fossil leaves and palynomorphs suggest a wet, subtropical habitat with a flora that included ferns, horsetails, cycads, conifers, and diverse and abundant angiosperms^45^. The diversity of the fossil vegetation suggests that the Kaiparowits dinosaurs had access to a great variety of plants, though this does not necessarily indicate high, year-round levels of acceptable forage. It is possible that seasonal or episodic intervals of inadequate browse drove some dinosaurs to utilize the celluloses and hemicelluloses available in rotted wood. This hypothesis infers that the rotting wood and invertebrates offered a diet of last resort. However it seems likely that shortages of adequate browse would have compelled dinosaurs to emigrate to other areas.

The converse hypothesis is that the ancient fecal producers actively exploited rotting wood to help fulfill specific nutritional needs that could not be satisfied by a more typical herbivore diet. In this scenario, decaying wood provided a cellulose-rich medium that also delivered a supply of proteinaceous fauna. This hypothesis for dietary motivation is consistent with the feeding behaviors of extant birds (avian dinosaurs). Adequate sources of protein and calcium are particularly important to breeding birds^47–49^ because requirements for protein are increased above maintenance levels to support ovary and oviduct development, as well as egg production^43^. Calcium demands are also elevated for egg-laying^50, 51^. Although medullary bone can provide a temporary source of calcium for egg-laying females, this only offers interim protection from calcium deficiencies^44^. Such nutritional needs can explain major changes in breeding bird diets^49^. Female birds can augment their protein intake by consuming more animal tissues when nesting^43, 47^, and often ingest calcium-rich materials such as mollusk shells, calcareous grit, or eggshells before laying eggs^51, 52^.

It is reasonable to infer that the non-avian dinosaurs that produced the Kaiparowits dung had similarly elevated nutritional requirements during breeding. The challenge of finding enough proteinaceous foods to satisfy the needs of a large-bodied dinosaur could have been met by capitalizing on the fairly predictable sources of animal protein in the invertebrate-rich community of rotting wood. This tactic is practiced by extant black bears that are able to consume large quantities of invertebrates by targeting rotting logs^53^.

There is no definitive evidence linking the Kaiparowits coprolites with reproductive activities. However the large, multi-deposit coprolite masses may provide some indirect support for correlation of reproductive activities with the woody coprolites, because latrine behavior (repeated defecation in a confined area) can be indicative of animals that are spatially constrained^54^. Nesting activities would have necessarily curtailed nomadic movements by breeding dinosaurs.

The hypothesis of decayed wood as a source of invertebrate fare during breeding was previously posited to explain the apparent stratigraphic association of the woody Two Medicine coprolites with *Maiasaura* nest sites. However, those coprolites showed no clear signs of active consumption of animal tissues^16^.

### Interpreting how and why crustaceans were consumed

The extensive fragmentation of the crustacean cuticle in the coprolites indicates that the crustaceans were ingested by the dinosaurian defecators, and could not have been post-depositional visitors to the fecal masses. The association of the cuticle with a preponderance of rotting wood suggests that consumption of the crustaceans occurred in a terrestrial environment. A number of extant crustaceans are known to frequent decaying logs^55, 56^, so it is likely that the consumed Cretaceous crustaceans displayed similar behavior and were accessible on land.

Three different scenarios can be posited to explain how crustaceans were consumed by the dinosaurian defecators: 1) through active hunting, 2) via targeted consumption of a resource patch that hosted abundant invertebrates (i.e., rotting wood), and 3) by inadvertent ingestion while feeding on other plant tissues. Scenario two represents a middle ground between active predation and indifferent consumption. It is based upon the invertebrate-rich nature of rotting wood, and the inference that the presence of crustacean cuticle in the coprolites indicates acceptance of crustaceans as acceptable or desirable foods. As such, the first two scenarios describe variations on the concept of “intentional” consumption. It is difficult to prove “intention” in the fossil record, but several factors bear on which of these schemas were most likely.

The most telling evidence is the fact that multiple examples of fragmented crustacean cuticle in the coprolites demonstrate recurring ingestion of crustaceans at different stratigraphic levels and at sites up to 20 km apart. This points to repeated crustacean consumption that occurred over a sizeable geographic area and over a period of time represented by ~100 meters of sediments.

The size of the crustaceans and the ability of the dinosaurs to control their intake of appropriate foods also bear on whether the crustaceans were intentionally or accidentally consumed. A 5-cm crustacean represents a significant proportion of the beak width of an ornithischian dinosaur. Mallon and Anderson^57^ found that the maximum beak widths of five genera (40 adult individuals) of hadrosaurs from the Dinosaur Park Formation ranged from ~8.0 to 24.8 cm. Individual crustaceans thus comprised ~20 to 60% of the width of common Campanian hadrosaurid beaks, suggesting that it is unlikely that the crustaceans were unwittingly swallowed. The selective feeding behavior of extant vertebrates supports this inference. Foodstuffs that induce satiation or fulfil nutritional needs are favored, while those that cause malaise are rejected^58–60^. These food responses have been correlated with the sensitivity of taste buds to compounds of nutritional importance (e.g., protein, digestible carbohydrates, calcium, and potential toxins). Indeed, both lizards^61^ and birds^61, 62^ have been observed to reject unacceptable foods that the animals had already taken into their mouths. Rejected foodstuffs are not necessarily toxic. One study documented that when canvasback ducks foraged underwater on a mixture pondweed tubers, corn, and insect larvae, they rapidly spit out the less desirable individual kernels of corn^62^. Although we do not know the oral processing capabilities of the dinosaurian defecators, extant phylogenetic bracketing^63^ suggests that they had some discriminatory capabilities and would not have repeatedly ingested foods that were not nutritionally useful.

The documented occurrence of crustacean cuticle in at least ten Kaiparowits coprolites, the substantial size of the crustaceans, and the selective feeding behavior of extant reptiles all suggest that the crustaceans were satisfactory or preferred food items for the dinosaurian defecators. Thus, the presence of crustaceans in the coprolites is best explained by the invertebrate-exploiting feeding behaviors laid out in scenarios one or two. Crustaceans sheltering in rotting wood would have been relatively easy to locate, but it is also possible that the dinosaurs ingested crustaceans that they encountered in other microhabitats. Nevertheless, it may be difficult to determine whether individual crustaceans were intentionally hunted.

### Phylogenetic context

**Figure 4:**
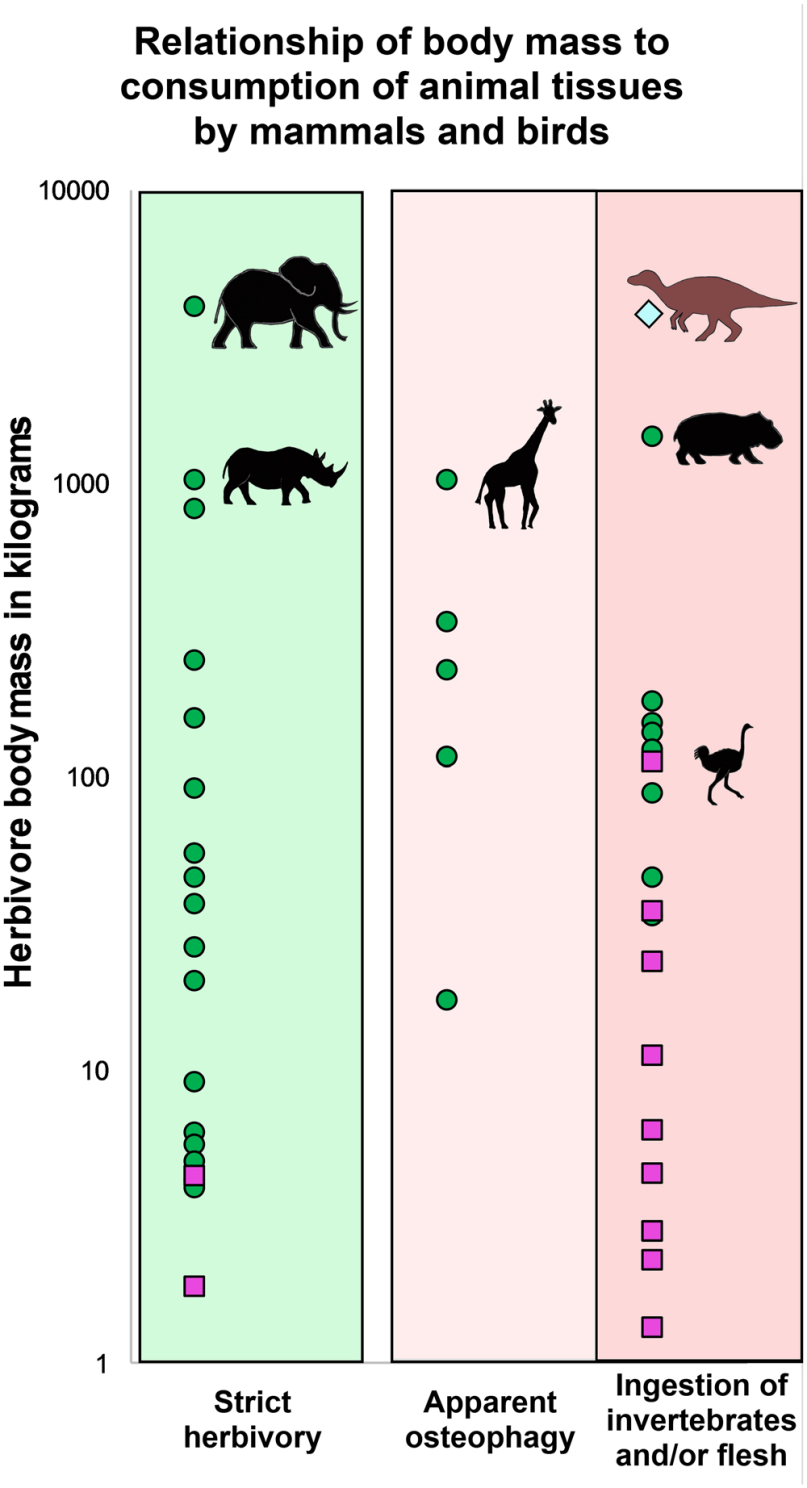
Graph depicting the relationship between vertebrate body mass and known consumption of animal tissues. Extant mammals are indicated by green squares, extant birds by pink dots, and the Kaiparowits defecators by a blue diamond. Data are based on literature sources documenting the body mass and feeding habits of 30 mammals and 12 birds that are primarily herbivorous (see Supplementary Data Table S2 and Supplementary References S3); no more than two members of each family are represented. Black silhouettes show four extant mammalian herbivores (African elephant, black rhinoceros, giraffe, and hippopotamus) and ostrich, the largest extant bird. Brown silhouette indicates possible Kaiparowits defecator, based on the estimated size of adult *Maiasaura* dinosaurs^73^.

Although the Kaiparowits defecators were probably ornithischian dinosaurs, an overview of the incidence of omnivory among extant avian dinosaurs (theropods) and mammalian herbivores can provide useful perspectives on the phylogenetic context of herbivore feeding patterns. The data in Fig. 4 were obtained from published descriptions of the feeding habits of a variety of primarily herbivorous extant vertebrates that are at least 1 kg in mass (a spreadsheet of the taxa, body masses, and diets can be found in Supplementary Data Table S2; the associated references are available in Supplementary References S3). We note that the boundary between herbivory and omnivory is somewhat artificial, because feeding habits reflect changing physiological needs and/or seasonal or geographic differences in resource availability. However, for this survey, 42 animal species from 24 mammalian and 11 avian families were characterized as demonstrating omnivorous behavior if several examples of their ingesting animal tissues have been documented. Two categories of omnivorous behavior are recognized: inferred osteophagy-driven ingestion of vertebrate bone, and consumption of invertebrates and/or vertebrate soft tissues. These categories are differentiated to help resolve dietary differences between animals that seek bones as a source of minerals from those that ingest animal tissues for protein.

There are far fewer clades of large herbivorous birds than herbivorous mammals; only ~3% of bird taxa feed primarily on green plant tissues^44^. Moreover, the largest extant avian body size of ~111 kg (ostrich) is considerably smaller than the body masses of mammalian megaherbivores (>1,000 kg) and many other ungulates. Nonetheless, it is notable that most (>80%) of the avian species represented in Fig. 3 are known to utilize at least some animal tissues. In contrast, many plant-eating mammals appear to be strictly herbivorous. Of the 30 herbivorous mammalian species listed in Fig. 3, fewer than half (~43%) are known to feed on animal tissues and more than half of this subset are reported to only eat bone. Such osteophagous feeding behavior has been reported in numerous ungulates^64^ and probably reflects a deficiency in phosphorus or (more rarely) calcium^65^ rather than a sought-after source of protein.

The distribution of omnivorous behavior among extant herbivores suggests that while feeding patterns are variable, animal foods are more consistently found in the diets of herbivorous birds than in the diets of herbivorous mammals. This mirrors differences in reproductive strategies. Gestation and lactation in large mammals last for extended periods of time, but oviparous vertebrates require a more rapid influx of calcium and protein to produce a clutch of eggs within a relatively short interval^66^.

The fact that the largest extant avian dinosaurs are an order of magnitude smaller than extinct ornithischians complicates comparisons of their respective diets. Nevertheless, the fossil evidence that some large herbivorous dinosaurs occasionally ingested sizeable invertebrates is congruent with the feeding habits of their extant avian relatives.

## Conclusions

Studies on the evolution of herbivory in non-avian dinosaurs suggest that highly derived herbivorous dinosaur taxa arose from basal forms that were carnivorous or omnivorous^11, 67^. If this evolutionary scenario holds, it is possible that many extinct and extant herbivorous dinosaurs retained some ancestral propensities to utilize animal foods. Indeed, other studies have suggested that dinosaurs that were previously interpreted as wholly herbivorous were in fact omnivorous^11, 16, 68, 69^. It is difficult to verify omnivory in the fossil record, however. Thus the Kaiparowits coprolites provide rare perspectives on dinosaur paleobiology and reveal complex and flexible feeding habits that are more consistent with the diets of extant birds than the strict herbivory of most mammalian megaherbivores. The multiple coprolites that record the ingestion of sizable and nutritionally-rich crustaceans suggest that this was not a random diet, but a regular–perhaps seasonal—feeding strategy. Although it is possible that the dinosaurs inadvertently ingested the crustaceans, this scenario would not only suggest a remarkable number of dietary coincidences, but that the dinosaurs lacked the ability to discriminate among potential foodstuffs. It is more likely that the dinosaurian defecators opportunistically exploited the crustaceans and other invertebrates in rotting wood. This resource patch would have offered a nutritious complex of plant polysaccharides that hosted dependable sources of protein and fulfilled specific nutritional needs—possibly related to life history strategies.

This evidence challenges simplistic interpretations of the feeding habits of megaherbivorous dinosaurs and serves to remind us that these animals have no modern analogs. No extant vertebrate clade integrates the combination of very large body masses, oviparity with high potential reproductive output^70^, multi-year maturation, and considerable time dedicated to reproductive activities^71^. The consumption of the complex wood and crustacean diet may well have been linked to reproductive activities, but more substantive evidence will be needed to test this hypothesis.

## Methods

The surfaces of hundreds of coprolite fragments from 15 sites were examined for recognizable cuticle fragments with a Leica MZ 12.5 stereo microscope. Fourteen thin sections from four samples were prepared and analyzed with a Leica DMR light microscope. Photomicrographs were made with a Canon 5D Mark II digital camera, and multiple images of portions of specimens were integrated with Zerene Stacker software to increase depth of field. Qualitative element maps were generated from two carbon-coated thin sections with a JEOL JXA-8600 electron microprobe at the University of Colorado Boulder. Uncoated coprolite specimens were also examined with a Hitachi TM 3030 tabletop SEM under low vacuum conditions at Kent State University. The coprolite fossils and thin sections are reposited in the collections of the Denver Museum of Nature and Science, and the fossil crab specimens from the Masuk Formation are catalogued in the Sam Noble Oklahoma Museum of Natural History. Data from the literature supporting Table 1 are available in the electronic supplementary materials.

## Electronic supplementary material

Supplementary Info S1 and S3

Supplementary Data Table S2
