# Supplementary material for: Consumption of crustaceans by megaherbivorous dinosaurs: dietary flexibility and dinosaur life history strategies
Source: Sci Rep. 2017 Sep 21;7:11163. doi: 10.1038/s41598-017-11538-w (PMC5608751; doi:10.1038/s41598-017-11538-w)
Supplement: Supplementary file 1 — Supplementary Info S1 and S3 [file 41598_2017_11538_MOESM1_ESM.pdf]

Chin, K., Feldmann, R.M., and Tashman, J.N. Consumption of crustaceans by megaherbivorous dinosaurs: dietary flexibility and dinosaur life history strategies

Supplementary Microprobe Maps S1:

Coprolite thin section electron microprobe elemental maps of DMNH EPV.62494, BP-12-13f (analyzed area in red box on photomicrograph below) generated with a JEOL JXA-8600 electron microprobe at the University of Colorado Boulder.

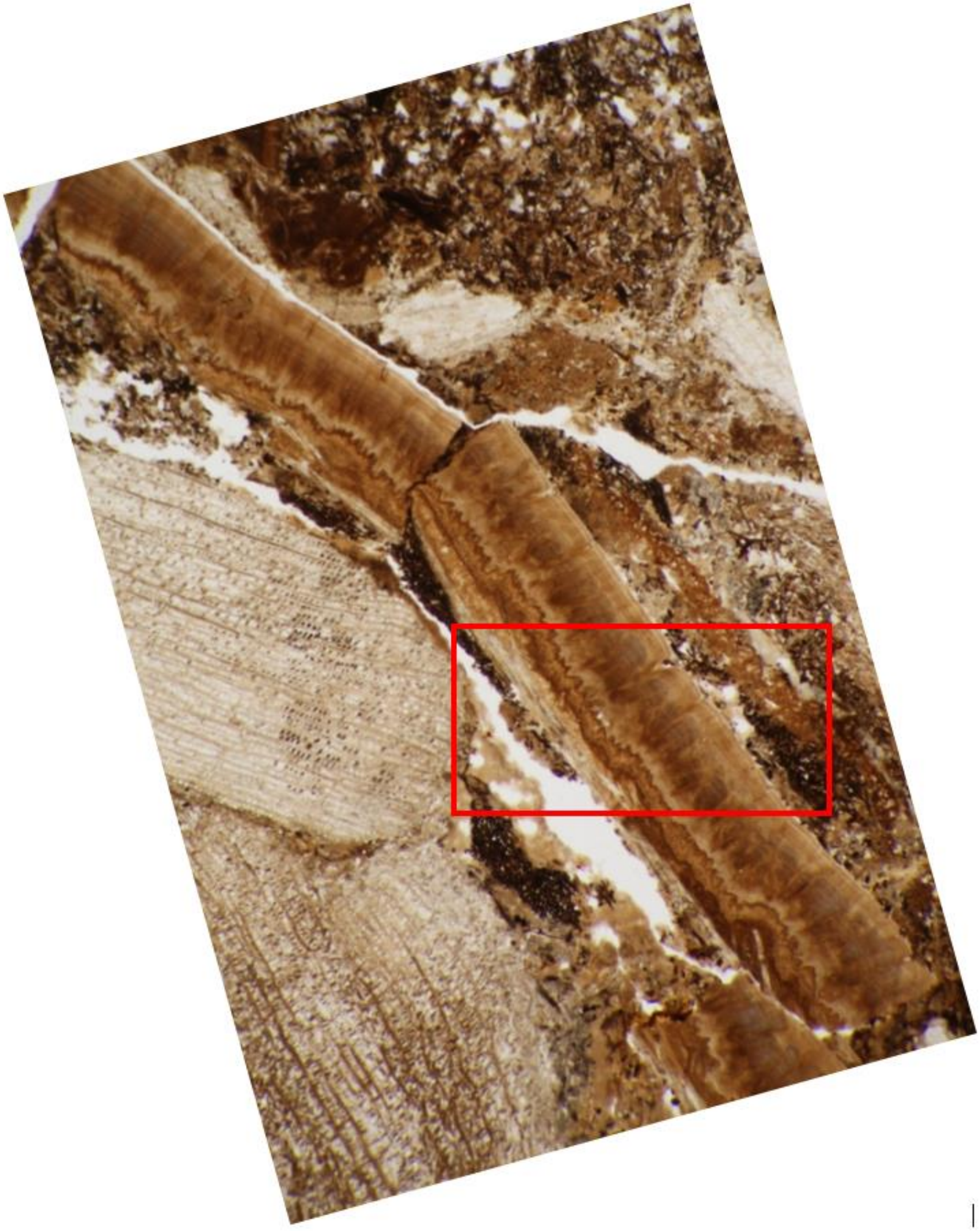

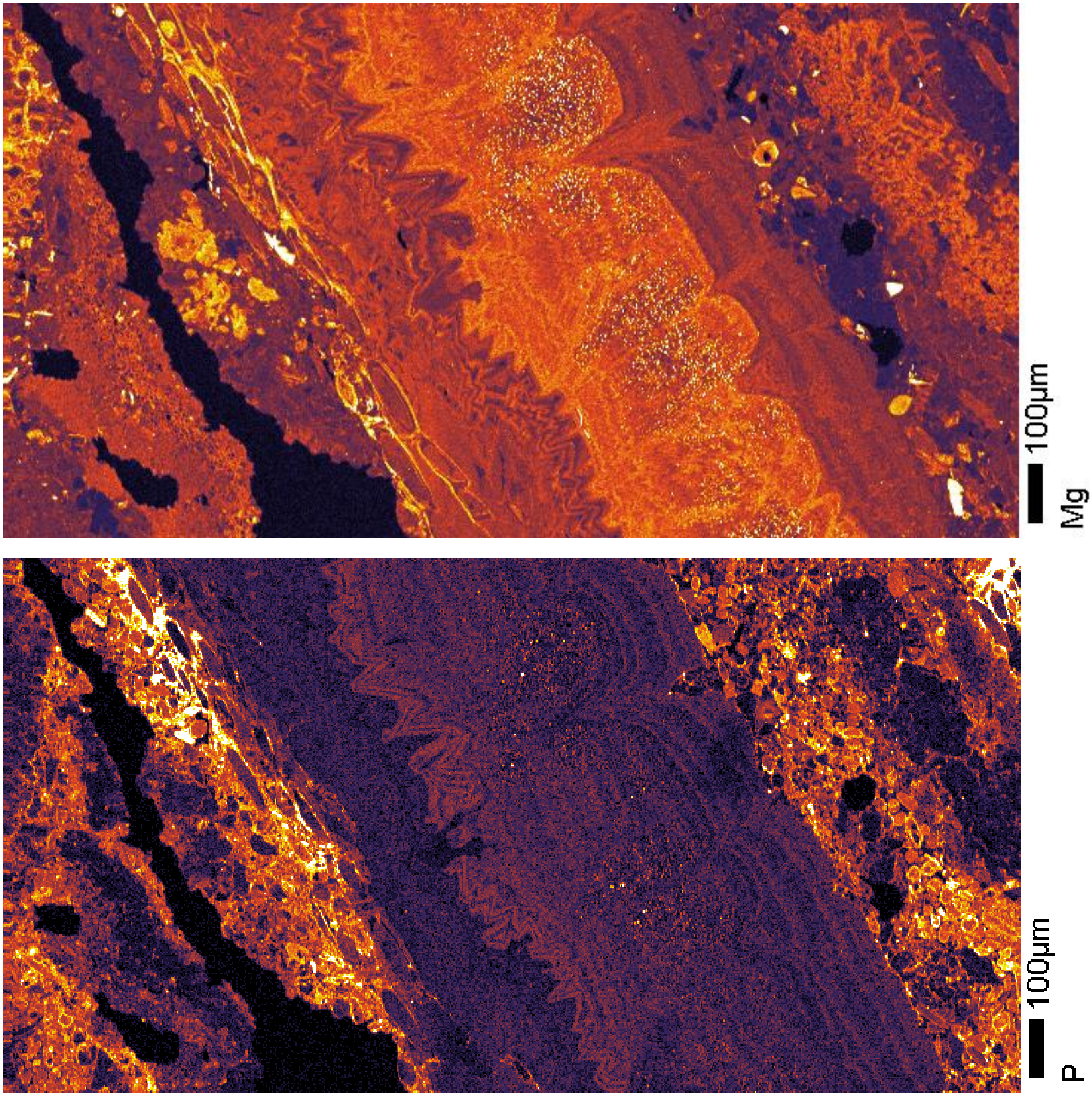
100µm
Mg
100µm
P

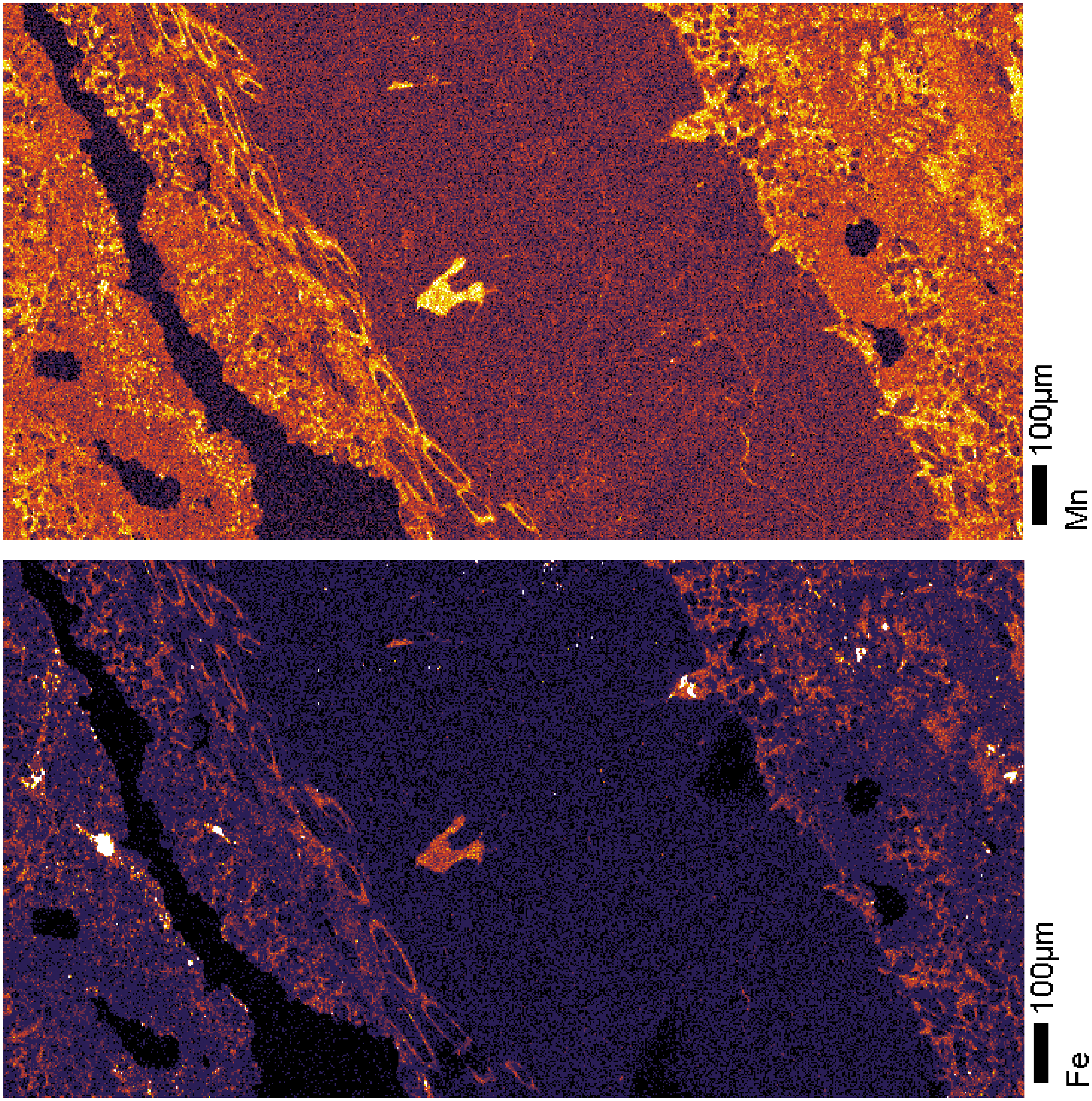
100µm
Mn
100µm
Fe

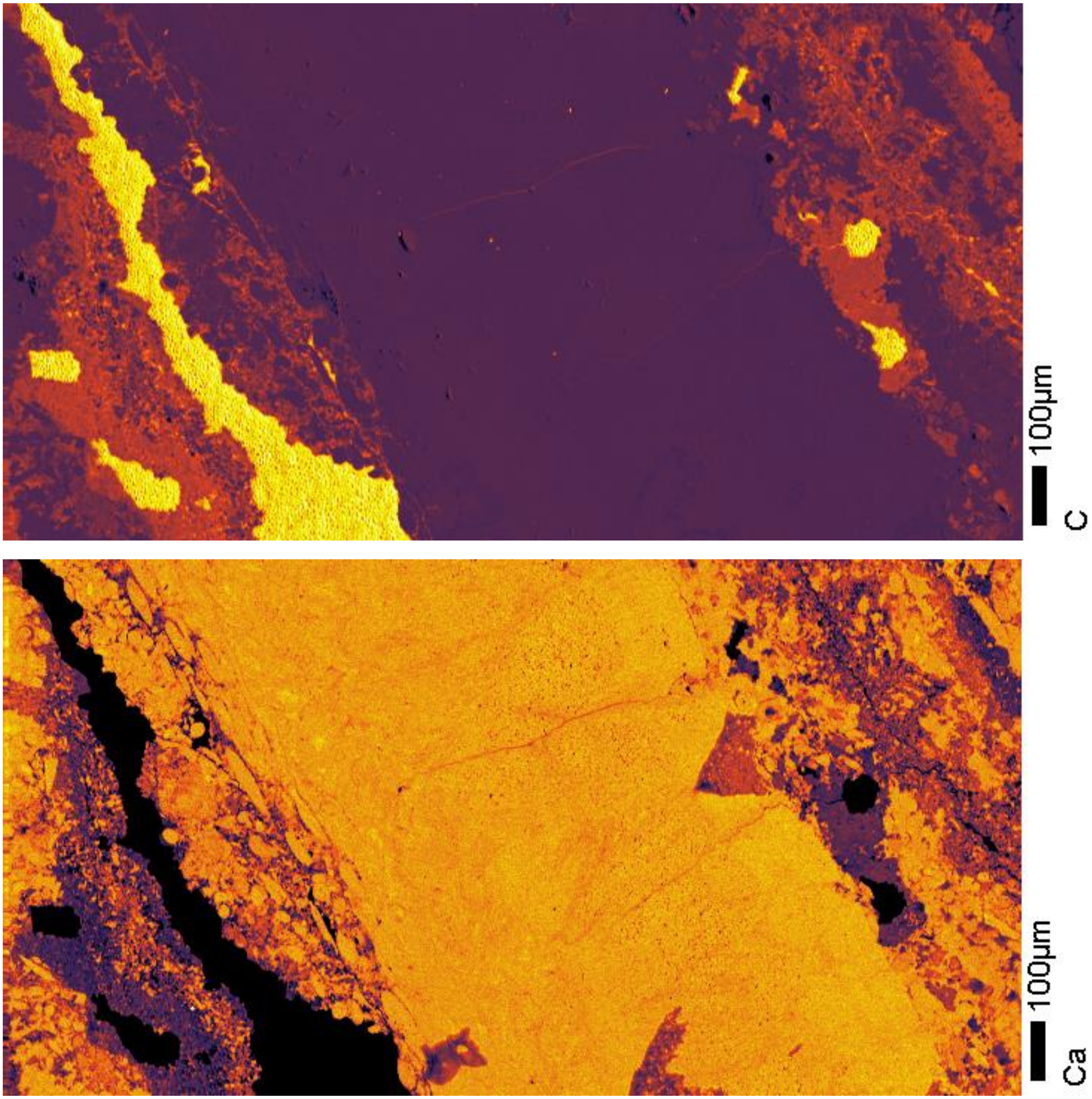
100µm
C
100µm
Ca

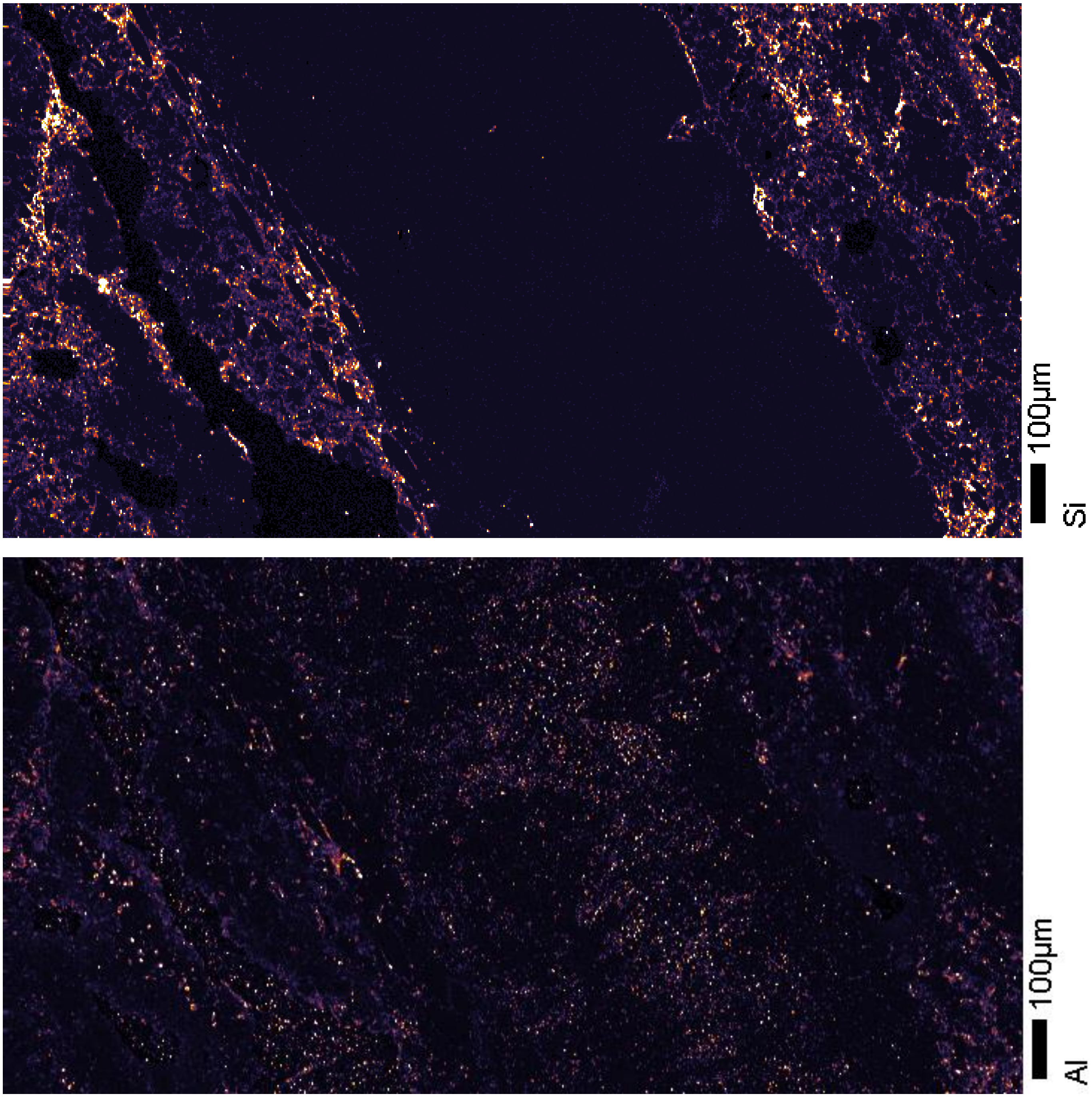
Si
100µm
Al
100µm

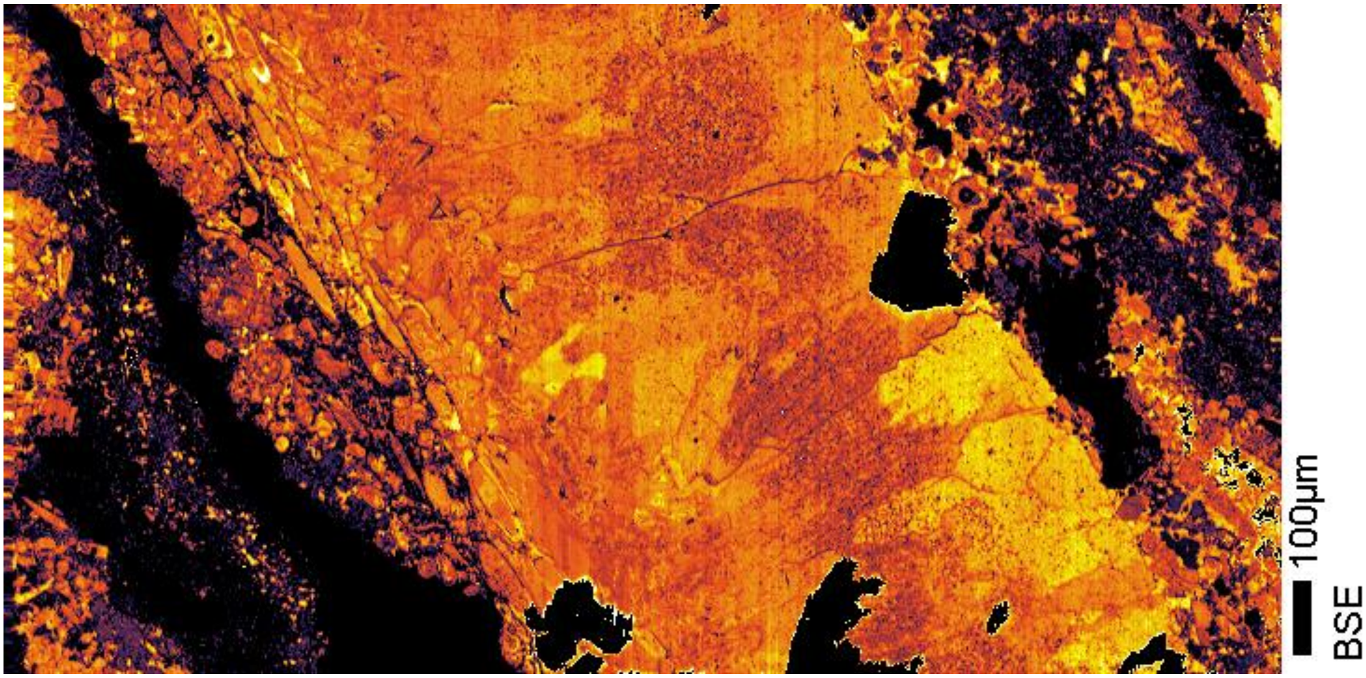
100μm
BSE

Supplementary References S3:
References cited in Supplementary Data Table S2 of herbivore body masses and degree of consumption of animal tissues

1. Agana, A. A., Aganga, A. O. & Omphile, U. J. Ostrich feeding and nutrition. *Pakistan J. Nutr.* **2,** 60–67 (2003).
2. Asensio, N., Cristobal-Azkarate, J., Dias, P. A. D., Vea, J. J. & Rodríguez-Luna, E. Foraging habits of *Alouatta palliata mexicana* in three forest fragments. *Folia Primatol.* **78,** 141–153 (2007).
3. Baber, M. J. & Craig, J. L. The relationship between foraging behavior and habitat use by South Island takahe (*Porphyrio hochstetteri*) on Tiritiri Matangi Island. *Notornis* **50,** 59–66 (2003).
4. Bahamonde, N., Martin, S. & Sbriller, A. P. Diet of guanaco and red deer in Neuquen Province, Argentina. *J. Range Manag.* **39,** 22–24 (1986).
5. Ballari, S. A. & Barrios-García, M. N. A review of wild boar *Sus scrofa* diet and factors affecting food selection in native and introduced ranges. *Mamm. Rev.* **44,** 124–134 (2014).
6. Banko, W. E. The trumpeter swan, its history, habits, and population in the United States. in *North American Fauna, Number 63* 214 (U.S. Fish and Wildlife Service, 1960).
7. Barreto, G. R., Hernandez, O. E. & Ojasti, J. Diet of peccaries (*Tayassu tajacu* and *T. pecari*) in a dry forest of Venezuela. *J. Zool.* **241,** 279–284 (1997).
8. Barreto, G. R. & Quintana, R. D. Foraging strategies and feeding habits of capybaras in *Capybara: Biology, Use and Conservation of an Exceptional Neotropical Species* (eds. Moreira, J. R., Ferraz, K. M. P. M. B., Herrera, E. A. & Macdonald, D. W.) (Springer Science and Business Media, 2013).
9. Barthelmess, E. L. *Hystrix africaeaustralis*. *Mamm. Species No. 788* 1–7 (2006).
10. Bodmer, R. E. Ungulate biomass in relation to feeding strategy within Amazonian forests. *Oecologia* **81,** 547–550 (1989).
11. Borges, L. V. & Colares, I. G. Feeding habits of capybaras (*Hydrochoerus hydrochaeris*, Linnaeus 1766), in the Ecological Reserve of Taim (ESEC - Taim) - South of Brazil. *Brazilian Arch. Biol. Technol.* **50,** 409–416 (2007).
12. Britt, A., Randriamandratonirina, N. J., Glasscock, K. D. & Iambana, B. R. Diet and feeding behavior of *Indri indri* in a low-altitude rain forest. *Folia Primatol.* **73,** 225–239 (2002).
13. Cadieux, M.-C., Gauthier, G. & Hughes, R. J. Feeding ecology of Canada geese (*Branta canadensis interior*) in sub-arctic inland tundra during brood-rearing. *Auk* **122,** 144–157 (2005).

14. Chalukian, S. C., de Bustos, M. S. & Lizarraga, R. L. Diet of lowland tapir (*Tapirus terrestris*) in El Rey National Park, Salta, Argentina. *Integr. Zool.* **8,** 48–56 (2013).
15. Chiarello, A. G. Diet of the Atlantic forest maned sloth *Bradypus torquatus* (Xenarthra: Bradypodidae). *J. Zool.* **246,** 11–19 (1998).
16. Dalke, P. D., Clark Jr., W. K. & Korschgen, L. J. Food habit trends of the wild turkey in Missouri as determined by dropping analysis. *J. Wildl. Manage.* **6,** 237–243 (1942).
17. Davies, S. J. J. F. The food of emus. *Aust. J. Ecol.* **3,** 411–422 (1978).
18. Dierenfeld, E. S., du Toit, R. & Braselton, W. E. Nutrient composition of selected browses consumed by black rhinoceros (*Diceros bicornis* in the Zambezi Valley, Zimbabwe. *J. Zoo Wildl. Med.* **26,** 220–230 (1995).
19. Dudley, J. P. Reports of carnivory by the common hippo *Hippopotamus amphibious*. *South African J. Wildl. Res.* **26,** 58–59 (1998).
20. Dunning Jr., J. *CRC Handbook of Avian Body Masses*. (CRC Press, 2008).
21. Estes, R. D. & Estes, R. K. The biology and conservation of the giant sable antelope, *Hippotragus niger variani* Thomas, 1916. *Proc. Acad. Nat. Sci. Philadelphia* **126,** 73–104 (1974).
22. Furness, R. W. Predation on ground-nesting seabirds by island populations of red deer *Cervus elaphus* and sheep *Ovis*. *J. Zool.* **216,** 565–573 (1988).
23. Gad, S. D. & Shyama, S. K. Studies on the food and feeding habits of gaur *Bos gaurus* H. Smith (Mammalia: Artiodactyla: Bovidae) in two protected areas of Goa. *J. Threat. Taxa* **1,** 128–130 (2009).
24. Gebert, C. & Verheyden-Tixier, H. Variations of diet composition of Red Deer (*Cervus elaphus* L.) in Europe. *Mamm. Rev.* **31,** 189–201 (2001).
25. Grant, T. A., Henson, P. & Cooper, J. A. Feeding ecology of trumpeter swans breeding in South Central Alaska. *J. Wildl. Manage.* **58,** 774–780 (1994).
26. Grobler, J. H. Feeding habits of the cape mountain zebra *Equus zebra zebra* Linn. 1758. *Koedoe* **26,** 159–168 (1983).
27. Hampton, P. D. The wintering and nesting behavior of the trumpeter swan. (University of Montana, 1981).
28. Hunt, H. E. & Slack, R. D. Winter diets of whooping and sandhill cranes in south Texas. *J. Wildl. Manage.* **53,** 11501154 (1989).
29. Jenkins, S. H. & Busher, P. E. *Castor canadensis*. *Mamm. Species No. 120* 1–8 (1979).

30. Knowlton, F. F. Food habits, movements and populations of moose in the Gravelly Mountains, Montana. *J. Wildl. Manage.* **24,** 162–164 (1960).

31. Larter, N. C. Seasonal changes in arctic hare, *Lepus arcticus*, diet composition and differential digestibility. *Can. Field-Naturalist* **113,** 481–486 (1999).

32. Lukianov, Y. Ecology of the Altai snowcock (*Tetraogallus altaicus*) in the Altai Mountains. *Gibier Faune Sauvag.* **9,** 633–640 (1992).

33. Madge, S. & McGowan, P. *Pheasants, Partridges, and Grouse: a Guide to the Pheasants, Partridges, Quails, Grouse, Guineafowl, Buttonquails, and Sandgrouse of the World*. (Princeton University Press, 2002).

34. Martella, M. B., Navarro, J. L., Gonnet, J. M. & Monge, S. A. Diet of greater rheas in an agroecosystem of Central Argentina. *J. Wildl. Manage.* **60,** 586–592 (1996).

35. Mattson, D. J., Blanchard, B. M. & Knight, R. R. Food habits of Yellowstone grizzly bears, 1977-1987. *Can. J. Zool.* **69,** 1619–1629 (1991).

36. Mayer, J. J. & Wetzel, R. M. *Catagonus wagneri*. *Mamm. Species No. 259* 1–5 (1986).

37. McLandress, M. R. & Raveling, D. G. Changes in diet and body composition of Canada geese before spring migration. *Auk* **98,** 65–79 (1981).

38. Meijaard, E., D'Huart, J. P. & Oliver, W. L. R. Family Suidae (pigs) in *Handbook of the Mammals of the World Volume 2. Hoofed Animals* (eds. Wilson, D. E. & Mittermeier, R. A.) 248–291 (Lynx Edicions, 2011).

39. Milton, S. J., Dean, W. R. J. & Siegfried, W. R. Food selection by ostrich in southern Africa. *J. Wildl. Manage.* **58,** 234–248 (1994).

40. Moore, B. D. & Foley, W. J. A review of feeding and diet selection in koalas (*Phascolarctos cinereus*). *Aust. J. Zool.* **48,** 317–333 (2000).

41. Nishida, T. & Uehara, S. Natural diet of chimpanzees (*Pan troglodytes schweinfurthii*): long-term record from the Mahale Mountains, Tanzania. *Afr. Study Monogr.* **3,** 109–130 (1983).

42. Novellie, P. A., Fourie, L. J., Kok, O. B. & van der Westhuizen, M. C. Factors affecting the seasonal movements of Cape mountain zebras in the Mountain Zebra National Park. *South African J. Zool.* **23,** 13–19 (1988).

43. Owen-Smith, R. N. *Megaherbivores: The influence of very large body size on ecology*. (Cambridge University Press, 1988).

44. Parker, G. R. Morphology, reproduction, diet, and behavior of the Arctic hare (*Lepus arcticus monstrabilis*) on Axel Heiberg Island, Northwest Territories. *Can. Field-Naturalist* **91,** 8–18 (1977).

45. Pereira, J. A., Quintana, R. D. & Monge, S. Diets of plains vizcacha, greater rhea and cattle in Argentina. *J. Range Manag.* **56,** (2003).

46. Powzyk, J. A. & Mowry, C. B. Dietary and feeding differences between sympatric *Propithecus diadema diadema* and *Indri indri*. *Int. J. Primatol.* **24,** 1143–1162 (2003).

47. Prevett, J. P., Marshall, I. F. & Thomas, V. G. Spring foods of snow and Canada geese at James Bay. *J. Wildl. Manage.* **49,** 558–563 (1985).

48. Raedeke, K. J. & Simonetti, J. A. Food habits of *Lama guanicoe* in the Atacama Desert of northern Chile. *J. Mammal.* **69,** 198–201 (1988).

49. Reinecke, K. J. & Krapu, G. L. Feeding ecology of sandhill cranes during spring migration in Nebraska. *J. Wildl. Manage.* **50,** 71–79 (1986).

50. Ridpath, M. G. The Tasmanian native hen. *Australian Natural History, Vol. XIV, No. 11* 346–350 (1964).

51. Ridpath, M. G. The Tasmanian native hen, *Tribonyx mortierii*. *CSIRO Wildl. Res.* **17,** 1–51 (1972).

52. Rishworth, C., McIlroy, J. C. & Tanton, M. T. Diet of the common wombat, *Vombatus ursinus*, in plantations of Pinus radiata. *CSIRO Wildl. Res.* **22,** 333–339 (1995).

53. Roger, E. & Nette, T. Osteophagia among moose of Cape Breton Highlands. *Nov. Outdoors* **12,** 18 (2003).

54. Schaller, G. B., Jinchu, H., Wenshi, P. & Jing, Z. *The Giant Pandas of Wolong*. (University of Chicago Press, 1985).

55. Sekulic, R. & Estes, R. D. A note on bone chewing in sable antelope in Kenya. *Mammalia* **41,** 537–539 (1977).

56. Silva, M. & Downing, J. A. *CRC Handbook of Mammalian Body Masses*. (CRC Press, 1995).

57. Squires, J. R. & Anderson, S. H. Trumpeter swan (*Cygnus buccinator*) food habits in the greater Yellowstone ecosystem. *Am. Midl. Nat.* **133,** 274–282 (1995).

58. Tevis Jr., L. Summer behavior of a family of beavers in New York state. *J. Mammal.* **31,** 40–65 (1950).

59. Wallestad, R. & Eng, R. L. Foods of adult sage grouse in central Montana. *J. Wildl. Manage.* **39,** 628–630 (1975).

60. Wann, J. M. & Bell, D. T. Dietary preferences of the black-gloved wallaby (*Macropus irma*) and the western grey kangaroo (*M. fuliginosus*) in Whiteman Park, Perth, Western Australia. *J. R. Soc. West. Aust.* **80,** 55–62 (1997).
61. Western, D. Giraffe chewing a Grant's gazelle carcass. *East Africa Wildl. J.* **9,** 156–157 (1971).
62. Williams, G. R. The takahe (*Notornis mantelli* Owen, 1848): a general survey. *Trans. R. Soc. New Zeal.* **88,** 235–258 (1960).
63. Wilson, D. J., Grant, A. D. & Parker, N. Diet of kakapo in breeding and non-breeding years on Codfish Island (Whenua Hou) and Stewart Island. *Notornis* **53,** 80–89 (2006).
64. Wyatt, J. R. Osteophagia in Masai giraffe. *East Africa Wildl. J.* **9,** 157 (1971).
65. Yamagiwa, J., Mwanza, N., Yumoto, T. & Maruhasi, T. Seasonal change in the composition of the diet of eastern lowland gorillas. *Primates* **35,** 1–14 (1994).
